# Targeting MUS81 promotes the anticancer effect of WEE1 inhibitor and immune checkpoint blocking combination therapy via activating cGAS/STING signaling in gastric cancer cells

**DOI:** 10.1186/s13046-021-02120-4

**Published:** 2021-10-08

**Authors:** Chengguo Li, Qian Shen, Peng Zhang, Tao Wang, Weizhen Liu, Ruidong Li, Xianxiong Ma, Xiangyu Zeng, Yuping Yin, Kaixiong Tao

**Affiliations:** 1 Department of Gastrointestinal Surgery, Union Hospital, Tongji Medical College, Huazhong University of Science and Technology, Wuhan, 430022 China; 2 Department of Oncology, Tongji Hospital, Tongji Medical College, Huazhong University of Science and Technology, Wuhan, 430030 China

**Keywords:** MUS81, WEE1, Immune checkpoint blocking, cGAS/STING pathway, Gastric cancer

## Abstract

**Background:**

Identification of genomic biomarkers to predict the anticancer effects of indicated drugs is considered a promising strategy for the development of precision medicine. DNA endonuclease MUS81 plays a pivotal role in various biological processes during malignant diseases, mainly in DNA damage repair and replication fork stability. Our previous study reported that MUS81 was highly expressed and linked to tumor metastasis in gastric cancer; however, its therapeutic value has not been fully elucidated.

**Methods:**

Bioinformatics analysis was used to define MUS81-related differential genes, which were further validated in clinical tissue samples. Gain or loss of function MUS81 cell models were constructed to elucidate the effect and mechanism of MUS81 on WEE1 expression. Moreover, the antitumor effect of targeting MUS81 combined with WEE1 inhibitors was verified using *in vivo* and *in vitro* assays. Thereafter, the cGAS/STING pathway was evaluated, and the therapeutic value of MUS81 for immunotherapy of gastric cancer was determined.

**Results:**

In this study, MUS81 negatively correlated with the expression of cell cycle checkpoint kinase WEE1. Furthermore, we identified that MUS81 regulated the ubiquitination of WEE1 via E-3 ligase β-TRCP in an enzymatic manner. In addition, MUS81 inhibition could sensitize the anticancer effect of the WEE1 inhibitor MK1775 in gastric cancer *in vitro* and *in vivo.* Interestingly, when MUS81 was targeted, it increased the accumulation of cytosolic DNA induced by MK1775 treatment and activated the DNA sensor STING-mediated innate immunity in the gastric cancer cells. Thus, the WEE1 inhibitor MK1775 specifically enhanced the anticancer effect of immune checkpoint blockade therapy in MUS81 deficient gastric cancer cells.

**Conclusions:**

Our data provide rational evidence that targeting MUS81 could elevate the expression of WEE1 by regulating its ubiquitination and could activate the innate immune response, thereby enhancing the anticancer efficacy of WEE1 inhibitor and immune checkpoint blockade combination therapy in gastric cancer cells.

**Supplementary Information:**

The online version contains supplementary material available at 10.1186/s13046-021-02120-4.

## Background

Gastric cancer is an aggressive and recalcitrant tumor; it is the fifth most frequently occurring cancer and the third leading cause of cancer-related deaths worldwide [1]. Although its outcome has been partly improved due to the standard treatment, including surgery and chemoradiotherapy, the five-year survival rate remains unsatisfactory [2, 3]. Trastuzumab, a Her-2-targeted primary monoclonal antibody, is approved by the FDA for advanced gastric cancer treatment [4]. Although trastuzumab treatment can lead to prolonged benefit in Her-2 positive gastric cancer patients, limited durable responses are available in these cases [5, 6]. Thus, it is essential to identify novel targets and reagents that can be applied in gastric cancer treatment [7, 8].

DNA damage and repair (DDR) abnormality-induced DNA damage accumulation leads to genomic instability, an underlying hallmark of cancer [9, 10]. Cancer cells harbor specific DDR deficiency, and thus, depend on a particular DDR regulator or pathway to deal with both endogenous and exogenous DNA damage for survival, thereby providing rational evidence to target these preponderance pathways with a more approchable therapeutic window in cancer [11–13]. Furthermore, recent studies revealed that DNA damage-inducing approaches, such as radiotherapy and small-molecule inhibitors targeting the DNA damage pathways, generate cytosolic dsDNA that can be recognized by the pattern-recognition receptor cyclic GMP-AMP synthase (cGAS) and activated STING pathway [14–16]. Activation of cGAS/STING triggers antigen-presenting cells, such as macrophages, to increase T cell proliferation and infiltration in the tumor microenvironment, thereby enhancing the therapeutic effect of immune checkpoint blockade (ICB); this makes the combination therapy of DDR-related agents and ICB a novel promising strategy for cancer treatment [17, 18]. The DDR abnormality differs from the cancer types, and the expression profile of DDR-related proteins needs to be further investigated [19, 20]; therefore, identifying appropriate DDR-related agents and targets that can be used with ICB in gastric cancer treatment remains a challenge.

Methyl methanesulfonate and ultraviolet-sensitive gene 81 (MUS81) is an important endonuclease that plays pivotal roles in genomic stability by modulating a series of DNA processes, such as replication, damage repair, and transcription [21–23]. In malignant diseases, its expression level and biological function differ depending on the tumor type [24, 25]. Our previous study demonstrated that MUS81 was overexpressed and it promoted tumor metastasis by regulating the expression of the epithelial–mesenchymal transition (EMT) marker ZEB1 in gastric cancer [26]; however, its therapeutic value as a predictive target needs to be further studied in gastric cancer. In the present study, we identified that MUS81 inhibition promotes the deubiquitination of WEE1, which is recognized as a key DNA damage checkpoint kinase. Eventually, WEE1 inhibition triggers gastric cancer cell-intrinsic innate immunity through the activation of the cGAS/STING pathway, and thus, potentiates the anticancer effect of ICB therapy in MUS81 deficient gastric cancer cells.

## Methods

### Clinical specimens

Gastric cancer specimens and matched adjacent normal tissues of 26 patients were obtained from the Department of Gastrointestinal Surgery of Union Hospital, Tongji Medical College, Huazhong University of Science and Technology. The specimens were fixed in 4% neutral formalin and then embedded in paraffin. Written informed consent was obtained from all patients. This study was approved by the Ethics Committee of Union Hospital, Tongji Medical College, Huazhong University of Science and Technology, and complied with the Helsinki Declaration.

### Cell culture

Human gastric cancer cell lines SGC7901, BGC823, AGS; mouse gastric cancer cell line MFC; and human embryonic kidney cell line HEK293T, were obtained from the National Collection of Authenticated Cell Cultures of China. Gastric cancer cells were cultured in RPMI-1640 medium (Gibco) supplemented with 10% fetal bovine serum (FBS, Gibco) and 1% penicillin–streptomycin. The HEK293T cells were cultured in Dulbecco’s Modified Eagle’s Medium with high glucose, 10% FBS, and 1% penicillin–streptomycin, in a moist incubator with 5% CO_2_ at 37 °C.

Mouse lymphocytes from the spleen were isolated after lysis with lymphocyte separation solution (Tbdscience). Bone marrow-derived dendritic cells (BMDCs) were prepared as described previously [27]. Briefly, C57 mice aged 6–8 weeks were sacrificed, and both femurs and tibias were incised to obtain the bone marrow. After grinding, the bone marrow was filtered, red blood cells were lysed, and the bone marrow cells were resuspended in RPMI-1640 medium containing GM-CSF (10 ng/mL; #AF-315-03, PeproTech), IL-4 (10 ng/mL; #214–14, PeproTech), and 10% FBS, and then incubated in a moist incubator with 5% CO_2_ at 37 °C. Fresh medium containing 10 ng/mL GM-CSF and 10 ng/mL IL-4 was added on day 4. Cells containing approximately 70% immature DCs presenting CD11c+, low levels of major histocompatibility complex (MHC) class II, and low CD40 and CD86 expression levels were harvested on day 7.

### Plasmid construction and transfection, and lentivirus infection

Wild-type MUS81 was cloned into pLVX3-IRES-puro for expression in mammals. The MUS81 point mutation plasmid was constructed using a polymerase chain reaction (PCR)-based site-directed mutagenesis method, and the ubiquitin plasmid tagged with His was obtained from Addgene. The plasmid sequences are listed in Supplementary Table 1. Plasmid transfection was performed using Neofect transfection reagent (Neofect Biotechnology) according to the manufacturer’s instructions.

Lentiviruses targeting MUS81 and control short hairpin RNAs were purchased from Genechem. MUS81 knockdown cells were generated according to the manufacturer’s instructions. The sequences of lentiviruses targeting MUS81 are summarized in Supplementary Table 2.

### RNA extraction and quantitative reverse transcription PCR (qRT-PCR)

Total RNA was extracted using RNAiso Plus* (#9109, Takara) and then reverse transcribed into cDNA using PrimeScript RT Master Mix (#RR036A, Takara). The cDNA of each sample was diluted and then used for qRT-PCR with TB Green Premix Ex Taq II (#RR820A, Takara) on a StepOne Plus Real-Time PCR System (Thermo Fisher Scientific) to detect the mRNA level of the target gene. The mRNA levels of specific genes were standardized using GAPDH, and the fold change was calculated using the comparative Ct method (2^−ΔΔCt^). The primers used for qRT-PCR in this study are listed in Supplementary Table 3.

### MTT assay

Cells in the logarithmic growth phase were digested and resuspended to adjust the cell density. The cells were seeded in 96-well plates at a density of 1000 cells per well. The supernatant was discarded before detection; then, 100 μL of cell culture medium containing 0.5 mg/μL MTT was added to the well and the plates were incubated in a 37 °C moist incubator containing 5% CO_2_ for 4 h. Next, to resolve crystallization, dimethyl sulfoxide (DMSO) was added and the solution was agitated for 20 min in a constant temperature shaker at 37 °C. The absorbance of each well was measured using a microplate reader at a wavelength of 490 nm.

### Clonogenic assay

Cells in the logarithmic growth phase were collected. One thousand cells were added to each well of a 6-well plate. Thereafter, cells were treated with different concentrations of MK1775 (purchased from Selleckchem). After 10 d of cultivation, the cells were fixed with 4% paraformaldehyde for 20 min and visually stained with crystal violet for 20 min. Finally, ImageJ was used to count the clones in the plates.

### Western blot analysis

The cells were collected and washed three times with precooled phosphate-buffered saline (PBS). The total protein was extracted using RIPA lysate (#R0278, Sigma), and the protein concentration was determined using a BCA kit (#P0012, Beyotime). The proteins were separated using sodium dodecyl sulphate–polyacrylamide gel electrophoresis (SDS-PAGE) and transferred to a polyvinylidene fluoride membrane (Millipore). Thereafter, the membrane was blocked with 5% skim milk for 1 h before incubation with the primary antibody. The membrane was incubated with the primary antibody overnight at 4 °C and then with the secondary antibody conjugated with horseradish peroxidase (HRP) for 1 h at room temperature. The membrane was imaged with ECL reagents (#6883, Cell Signaling Technology) using the Invitrogen iBright CL1000 imaging system (Thermo Fisher Scientific). The primary antibodies included anti-MUS81 (1:1000; #ab 247,136, Abcam), anti-WEE1 (1:1000; #13084, Cell Signaling Technology), anti-Flag (1:1000; #14793, Cell Signaling Technology), anti-β-TRCP (1:200; #sc-390,629, Santa Cruz), anti-ubiquitin (1:1000; #58395, Cell Signaling Technology), anti-Ki-67 (1:1000; #A00254, Boster Biological Technology), anti-p-TBK1 (1:1000; #5483, Cell Signaling Technology), anti-TBK1 (1:200; #sc-398,366, Santa Cruz), and anti-GAPDH (1:3000; #60004–1-Ig, Proteintech). The secondary antibodies included HRP-conjugated goat anti-rabbit (1:3000; #SA00001–15, Proteintech) and anti-mouse (1:3000; #SA00001–1, Proteintech).

### Enzyme-linked immunosorbent assay (ELISA)

ELISA testing for TNF-α (#1217202, DAKEWE), IL-1β (#1210122, DAKEWE), and IL-6 (#1210602, DAKEWE) was conducted following the manufacturer’s instructions. Briefly, 100 μL of cytokine standard or coculture supernatant was added to the precoated wells. Thereafter, 50 μL of biotinylated antibody was added and plates were incubated at 37 °C for 90 min. After washing, 100 μL streptavidin-HRP was added to each well and plates were incubated at 37 °C for 30 min. Finally, the wells were washed, and 100 μL TMB was added to each well. After incubation at 37 °C for 15 min, the reaction was stopped, and the absorbance was read at a wavelength of 450 nm.

### Ni-NTA pull-down

The His-Ub or Flag-MUS81 plasmid was transfected into HEK293T cells, and the cells were harvested after treatment with MG132 for 4 h. Thereafter, the cells were washed twice with PBS and then lysed with urea lysis that contained 8 M urea, 0.1 M NaH_2_PO_4_, 300 mM NaCl, and 0.01 M Tris (pH 8.0). The lysates were ultrasonicated to shear the DNA; 40 μL was taken as the input and the remaining lysates were incubated with Ni-NTA agarose beads (MCE) for 2 h at room temperature. The beads were then washed five times with urea lysis. After boiling and denaturing with loading buffer, the beads and input were subjected to SDS-PAGE.

### Co-immunoprecipitation

After 48 h of transfection with the corresponding plasmid, AGS cells were collected and lysed with lysis buffer containing 20 mM Tris (pH 7.5), 150 mM NaCl, 1% Triton X-100, and 1% protease inhibitor cocktail (#B14001, Bimake). The whole-cell lysate was centrifuged, the supernatant was discarded, 2 μg of antibody was added and the cell lysate was incubated overnight at 4 °C on a rotating shaker. Then, 30 μL of protein A/G magnetic beads (#B23201, Bimake) was added to the lysates and incubated for 2 h. The magnetic beads were washed five times with lysis solution. After boiling and denaturation, both beads and input were subjected to SDS-PAGE.

### Immunohistochemistry (IHC)

Tissue sections cut from the paraffin blocks were deparaffinized with xylene and rehydrated with gradient alcohol. Tissue sections were subjected to antigen repair after blocking the activity of endogenous peroxidase with 3% H_2_O_2_. In addition, the non-specific antibody binding sites were blocked using bovine serum albumin. The sections were then incubated with primary antibody (anti-MUS81 1:200; anti-WEE1 1:200; anti-Ki-67 1:300) and the corresponding secondary antibody, and subsequently stained with the SABC kit (#SA1054, Boster Biological Technology) according to the manufacturer’s instructions.

The IHC scores were calculated as described previously [26]. The staining intensity scores were as follows: 0 (negative), 1 (weak), 2 (medium), and 3 (strong); staining area scores were 0 (none), 1 (1–25%), 2 (26–50%), 3 (51–75%), and 4 (76–100%). The two scores were then multiplied to obtain the final IHC score. The number of positive cells was counted using the ImageJ software.

### Picogreen staining

The Quant-iT PicoGreen dsDNA Assay Kit (#P11496) was purchased from Thermo Fisher Scientific. For Picogreen staining, cells were incubated in a culture medium containing PicoGreen at a concentration of 3 μL/mL for 1 h at 37 °C. After fixing with 4% paraformaldehyde for 20 min and counterstaining with DAPI, the cells were photographed using a fluorescence microscope.

### Multiplexed immunofluorescence

For multiplexed immunofluorescence, the tissue sections were routinely deparaffinized, repaired with antigen, and blocked with donkey serum. They were then incubated with the primary antibody (1:500, #ab213524, PD-L1, Abcam; 1:400, #55397, CD8, Cell Signaling Technology; 1:500, # 14580–1-AP, perforin, Proteintech) overnight at 4 °C, followed by incubation with the corresponding HRP-labeled secondary antibody and fluorescence enhancer. After counterstaining with DAPI, the sections were scanned using Pannoramic MIDI (3DHISTECH).

### Bioinformatics analysis

First, the mRNA expression profile data of gastric cancer and corresponding paracancerous tissues (GSE62254) were downloaded from the Gene Expression Omnibus (GEO) database. https://www.ncbi.nlm.nih.gov/geo/. The standardization and transformation of GSE62254 were completed using the “affy” package in R4.0.0. Next, the Limma package was used for detecting the difference in mRNA expression between gastric cancer tissues and paracancerous tissues, and the results were visualized as a heatmap using the “pheatmap” package. Further analysis was conducted to analyze the expression of hub genes related to DNA damage repair, and then a volcano map was drawn. The patients were divided into two groups based on MUS81 mRNA level quartiles: MUS81 high expression and low expression groups. Overall survival (OS) analysis was conducted to explore the relationship between MUS81 and survival using the R package “survival”. The correlation between MUS81 and WEE1 was described using the Pearson coefficient. Gene Ontology (GO) enrichment analysis was performed to identify the potential functions of and diseases highly correlated with MUS81. Gene set enrichment analysis was further used to analyze the possible functions of MUS81-related differential genes.

### In vivo assays

The in vivo assays were approved by the Ethics Committee of Union Hospital, Tongji Medical College, Huazhong University of Science and Technology. For the gastric cancer nude mice model, 1.5 × 10^6^ SGC-7901 shScramble or shMUS81 cells in 100 μL PBS were subcutaneously injected into the right flank of Balb/c nude mice (purchased from HUAFUKANG Bioscience). One week later, the mice were randomly divided into four groups: control, shMUS81, MK1775, and shMUS81 plus MK1775 groups. MK1775 was administered via oral gavage at a dose of 20 mg/(kg.day). The body weight and tumor volume were measured every 2 d, and the tumor volume was calculated using a simple formula [(length × width × width)/2]. After 16 d of treatment, mice were euthanized on reaching an endpoint as per the Institutional Animal Care and Use Committee (IACUC) guidelines. The transplanted tumor was fixed with 4% paraformaldehyde and embedded in paraffin.

For the gastric cancer syngeneic model, 1 × 10^6^ mouse gastric cancer cell MFCs (shScramble or shMUS81) were subcutaneously injected into the right flank of 615 mice (purchased from HUAFUKANG Bioscience). One week later, the mice were randomly divided into eight groups: shScramble group, shScramble plus MK1775 group, shScramble plus PD-L1 antibody group, shScramble plus MK1775 and PD-L1 antibody group, shMUS81 group, shMUS81 plus MK1775 group, shMUS81 plus PD-L1 antibody group, shMUS81 plus MK1775, and PD-L1 antibody group. PD-L1 antibody was purchased from BioXCell and administered via intraperitoneal injection at a dosage of 200 μg every 3 d. MK1775 was orally administered at a dosage of 20 mg/(kg day). Bodyweight and tumor volume was measured every 2 d, and the tumor volume was calculated as (length × width × width)/2. The mice were sacrificed after 22 d of treatment.

### Statistical analysis

SPSS 25.0 (IBM SPSS Statistics, Armonk, NY) and GraphPad Prism 9.0 (GraphPad Software, Inc., La Jolla, CA) were used for statistical analysis. All results are expressed as the mean ± standard deviation (SD). For normally distributed data, the Student’s *t*-test was applied to compare differences between two groups, whereas the Mann-Whitney test was used for comparison of differences in the remaining cases. One-way analysis of variance was used to analyze the differences between multiple groups. The Pearson coefficient described the correlation of IHC scores between MUS81 and WEE1. Statistical significance was set at *P* < 0.05.

## Results

### MUS81 overexpression is linked to poor prognosis and WEE1 deficiency in gastric cancer

**Fig. 1:**
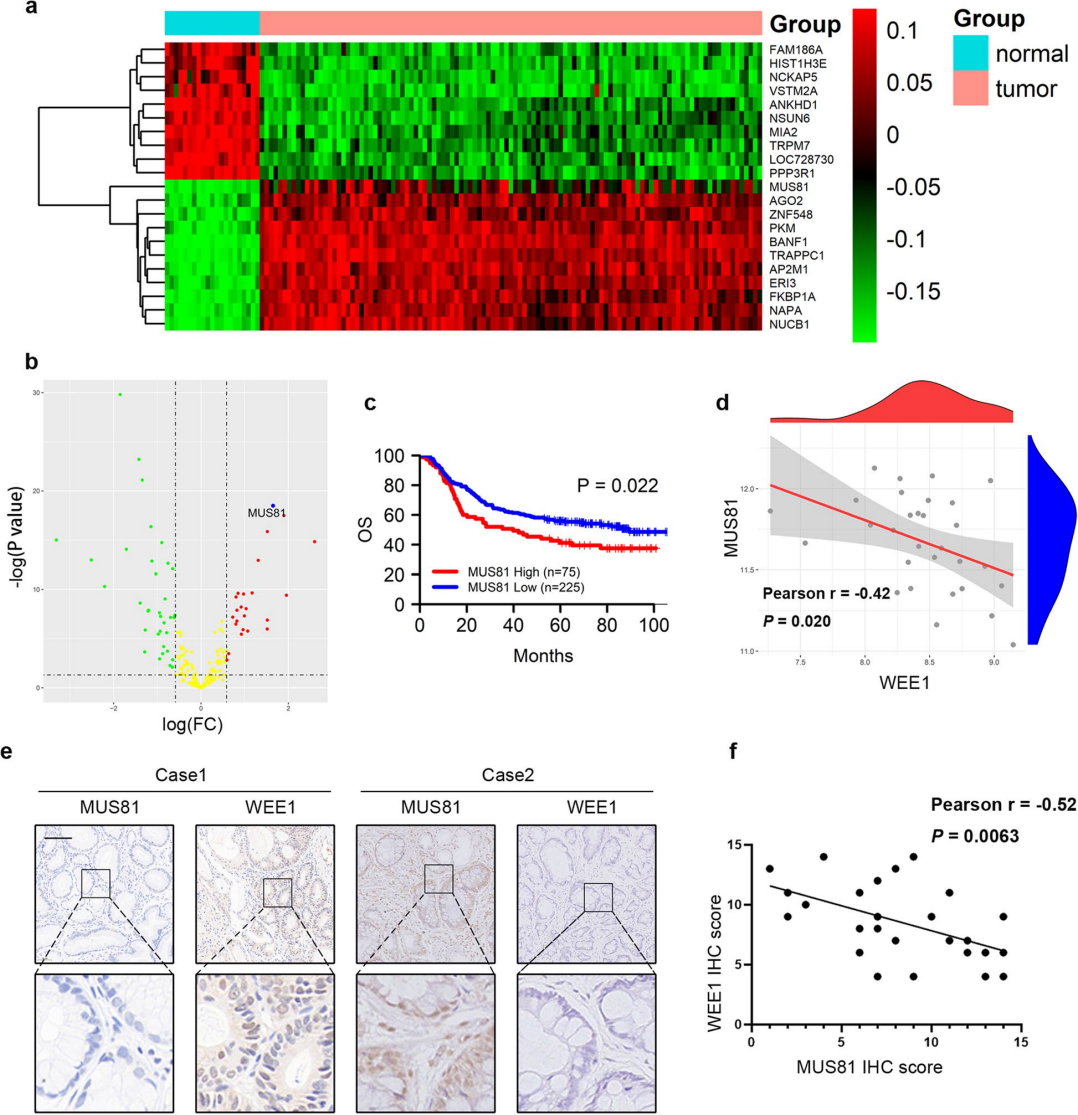
MUS81 overexpression links with poor prognosis and WEE1 deficiency in gastric cancer. **a** Heatmap of differentially expressed genes (DEGs) between gastric cancer and paracancerous tissues in the GSE62254 data set. The top 10 upregulated genes and top 10 downregulated genes are shown. **b** Volcano map of DEGs related to DNA damage and repair. The DEGs with ∣log (FC)∣ > 0.05 and -log (*P*-value) >1 are indicated; red, green, and yellow dots represent upregulated DEGs, down-regulated DEGs, and DEGs with non-significant changes, respectively. **c** High expression of MUS81 is associated with short overall survival time in patients with gastric cancer (GSE62254 data set). **d** Pearson correlation analysis of MUS81 and WEE1 in the GSE62254 data set. **e** Representative immunohistochemical staining of MUS81 and WEE1 in patients with gastric cancer. Scale bar: 100 μm. **f** Pearson correlation analysis was performed to validate the correlation of MUS81 and WEE1 protein expression in 26 patients with gastric cancer from Wuhan Union Hospital

We systematically investigated the altered expression of the molecules involved in the DDR network to identify potential targets that might be applied in gastric cancer treatment. First, we analyzed the differentially expressed genes (DEGs) in gastric cancer and paracancerous tissues using RNA-seq data obtained from the GEO database (GSE62254) (Fig. 1a). We then screened the genes related to DDR among the DEGs, further analyzed changes in their expression, and found that MUS81 was significantly overexpressed in patients with gastric cancer (Fig. 1b). Patients with gastric cancer with higher MUS81 levels had a poorer prognosis than patients with lower MUS81 expression (Fig. 1c). Based on the critical role and prognostic value of MUS81 in gastric cancer, we attempted to detect the signaling regulation network of MUS81. By analyzing the correlation between MUS81 and other vital DDR-related targets, we found that MUS81 was negatively correlated with WEE1 kinase expression (Pearson r = −0.42, *P* = 0.020) (Fig. 1d). Furthermore, we performed IHC staining of specimens from a cohort of 26 patients with gastric cancer and observed a significant negative correlation between MUS81 and WEE1 kinase expression (Pearson r = −0.52, *P* = 0.0063) (Fig. 1e and f). These data indicate the clinical relevance of WEE1 suppression in gastric cancer patients with MUS81 overexpression.

### MUS81 regulates β-TRCP-mediated WEE1 ubiquitination

**Fig. 2:**
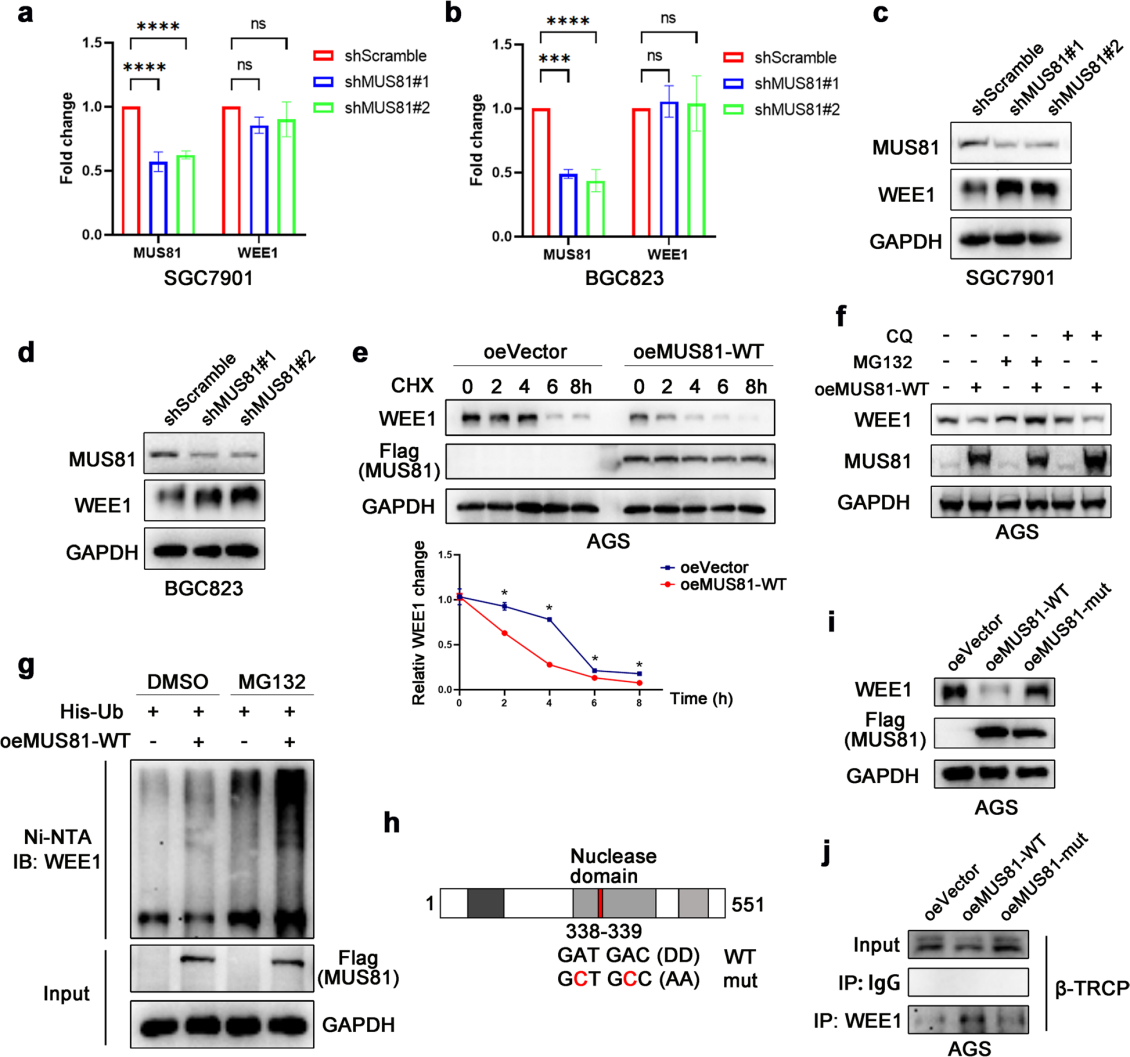
MUS81 regulates β-TRCP-mediated WEE1 ubiquitination. **a** and **b** PCR analysis of MUS81 and WEE1 after MUS81 knockdown in gastric cancer cell lines SGC7901 and BGC823. Data are presented as the mean ± SD (n = 3). **c** and **d** Western blot analysis of the impact of MUS81 knockdown on the expression of WEE1 at the protein level in gastric cancer cell lines SGC7901 and BGC823. **e** Representative images of WEE1 protein degradation after exposure of wild-type MUS81 (MUS81-WT) overexpression and parental AGS cells to cycloheximide (CHX; 50 μg/mL) for different time periods (top) and statistical line graph (bottom). Data are presented as the mean ± SD (n = 3). **f** Western blot analysis of the effect of chloroquine (10 μM) or MG132 (10 μM) on the degradation of WEE1 protein in MUS81-WT overexpression and parental AGS cells. **G** Ni-NTA pulldown analysis of 293 T cells transfected with the indicated plasmid to evaluate the effect of MUS81 on the ubiquitination of WEE1. His-Ub, His-tagged ubiquitin; oeMUS81-WT, FLAG-tagged wild-type MUS81; IB WEE1, immunoblotting with anti-WEE1 antibody. **h** Sketch map of MUS81 enzymatic mutant plasmid construction. Site-directed mutagenesis was used to replace two aspartic-acid residues within the MUS81 nuclease domain (338 and 339) with alanine residues. **i** Immunoblot of AGS cells transfected with the indicated plasmid, oeVector, FLAG-tagged wild-type MUS81 (oeMUS81-WT), and FLAG-tagged enzymatically inactivated MUS81 (oeMUS81-mut). **j** Co-immunoprecipitation was performed to detect the effect of MUS81 on the binding of E-3 ligase β-TRCP and WEE1

Next, we generated two MUS81 stable knockdown gastric cancer cell lines (SGC7901 and BGC823). The expression of WEE1 showed limited change at the transcription level (Fig. 2a and b), but consistent with our observations in gastric cancer tissues, the WEE1 protein level was significantly elevated in MUS81 knockdown cells (Fig. 2c and d). As described previously, AGS cells showed low expression of MUS81 [26]. We further constructed the MUS81 wild-type (MUS81-WT) plasmid and transfected it into AGS cells (oeMUS81-WT), whereas the control group was transfected with the vector plasmid (oeVector). Then, we treated AGS oeMUS81-WT and oeVector cells with cycloheximide to detect the half-time of WEE1 protein degradation. As shown in Fig. 2e, WEE1 protein was decreased in MUS81-WT cells compared with that in parental cells. After cycloheximide exposure for different time periods, compared with MUS81 overexpression cells, the parental cells exhibited delayed WEE1 degradation, indicating a long WEE1 degradation half-time (Fig. 2e). These data supported that MUS81 has an evident influence on the degradation of WEE1 protein. To further determine the mechanism of WEE1 degradation, we overexpressed wild type MUS81 in AGS cells and treated them with the autophagy inhibitor chloroquine or proteasome inhibitor MG132; MG132 but not chloroquine, exhibited a potent rescue effect on WEE1 protein degradation (Fig. 2f), suggesting that MUS81 might regulate WEE1 protein degradation in a ubiquitinated manner. As expected, the ubiquitination of WEE1 protein was significantly increased in MUS81-WT-overexpressing 293 T cells (Fig. 2g). Furthermore, we investigated whether this function is related to the enzymatic activity of MUS81. Site-directed mutagenesis was used to replace two aspartic-acid residues with alanine residues within the MUS81 nuclease domain (338 and 339), and then, a MUS81 enzymatic activity mutant (MUS81-mut) plasmid was constructed (Fig. 2h). As shown in Fig. 2i, overexpression of MUS81-WT, but not MUS81-mut, decreased the expression of WEE1 protein in the AGS cell line. To further investigate how MUS81 regulates WEE1 ubiquitination, we examined the effect of MUS81-WT or MUS81-mut overexpression on the binding of WEE1 and E-3 ligase β-TRCP, which specifically induced WEE1 protein degradation. As shown in Fig. 2j, overexpression of MUS81-WT, but not MUS81-mut, increased the binding of β-TRCP and WEE1 (Fig. 2j), thus decreasing the ubiquitination of WEE1 kinase. These data indicate that MUS81 regulates WEE1 protein levels post-transcriptionally in an enzymatic activity-dependent manner.

### MUS81 targeting sensitizes the anticancer effect of WEE1 inhibitor MK1775 in gastric cancer *in vitro* and *in vivo*

**Fig. 3:**
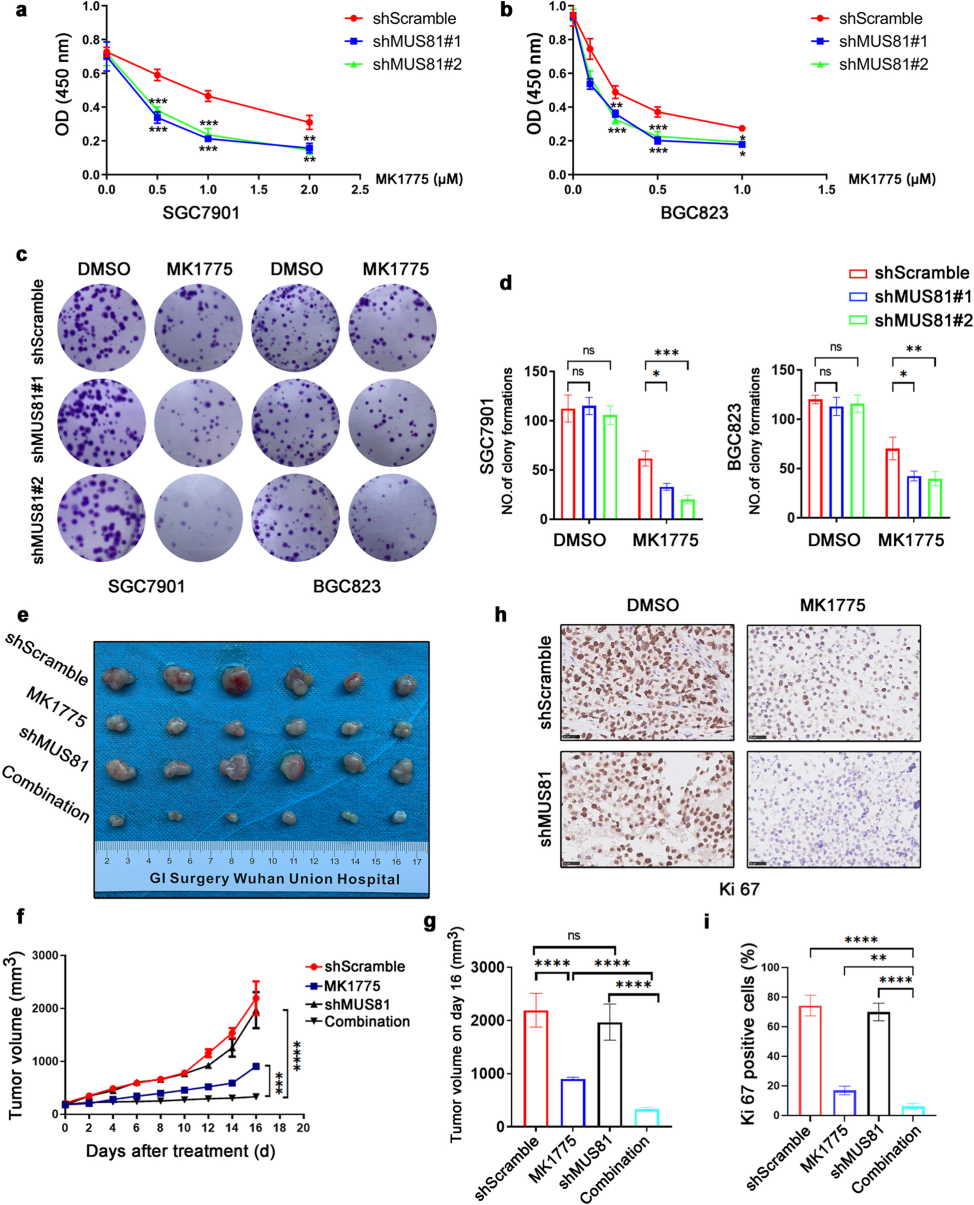
Targeting MUS81 sensitizes the anticancer effect of WEE1 inhibitor MK1775 in gastric cancer *in vitro* and *in vivo.* **a** and **b** MTT assay was performed to examine the effect of MK1775 on proliferation of MUS81 knockdown and parental gastric cancer cells SGC7901 and BGC823. Data are reported as mean ± SD for five independent experiments. **c** Clonogenic assay was performed to detect the anticancer effect of MK1775 in MUS81 knockdown and parental gastric cancer cells SGC7901 (left) and BGC823 (right). Representative images are displayed. **d** Data of the clonogenic assay in SGC7901 (left) and BGC823 (right) cells are reported as mean ± SD for three independent experiments. **e** The image of tumors in the SGC7901 gastric cancer xenograft mice model (n = 6 for each group) after treatment for 16 d. **f** Tumor growth curve of indicated groups in the SGC7901 gastric cancer xenograft mice model. The data are presented as mean ± SD (n = 6 for each group). **g** Tumor volume on day 16 after treatment. Data are presented as mean ± SD (n = 6 for each group). **h** Representative images of immunohistochemical (IHC) staining of Ki67 in the gastric cancer xenograft mice model. Scale bar: 25 μm. **i** IHC staining analysis of Ki 67-positive cells in different groups. The data are presented as mean ± SD (n = 3). * *P* < 0.05; ** *P* < 0.01; *** *P* < 0.001; **** *P* < 0.0001; *ns.*, not significant

Our observations led us to next evaluate whether targeting MUS81 could elevate the anticancer effect of WEE1 inhibitors in gastric cancer cells. Therefore, we performed the MTT assay, and analyzed the data to establish the dose-inhibition efficiency curves and calculated the IC_50_ of the WEE1 inhibitor MK1175 in different groups. As shown in Fig. 3a and b, MK1775 potently suppressed cell proliferation in a concentration-dependent manner and had a lower IC_50_ value in MUS81 knockdown gastric cancer cells than in MUS81 wild-type cells, and the 2D colony formation assay also showed less cell colony formation in MUS81 knockdown cells after MK1775 exposure (Fig. 3c and d). To further confirm the sensitization effect of MUS81 targeting, we generated the MUS81 knockdown and parental xenograft gastric cancer models. After treatment with MK1775 or solvent for 16 d, tumor volume was significantly decreased in the MUS81 knockdown group (Fig. 3e–g). In addition, IHC results indicated that after treatment with MK1775, the xenograft tumors in the MUS81 knockdown group had a low proliferative activity with a low Ki 67 index (Fig. 3h and i). Collectively, our results demonstrated that targeting MUS81 promoted the anticancer effect of MK1775 significantly *in vitro* and *in vivo.*

### MUS81 inhibition amplifies cytosolic DNA accumulation and promotes innate immune activation in gastric cancer cells

**Fig. 4:**
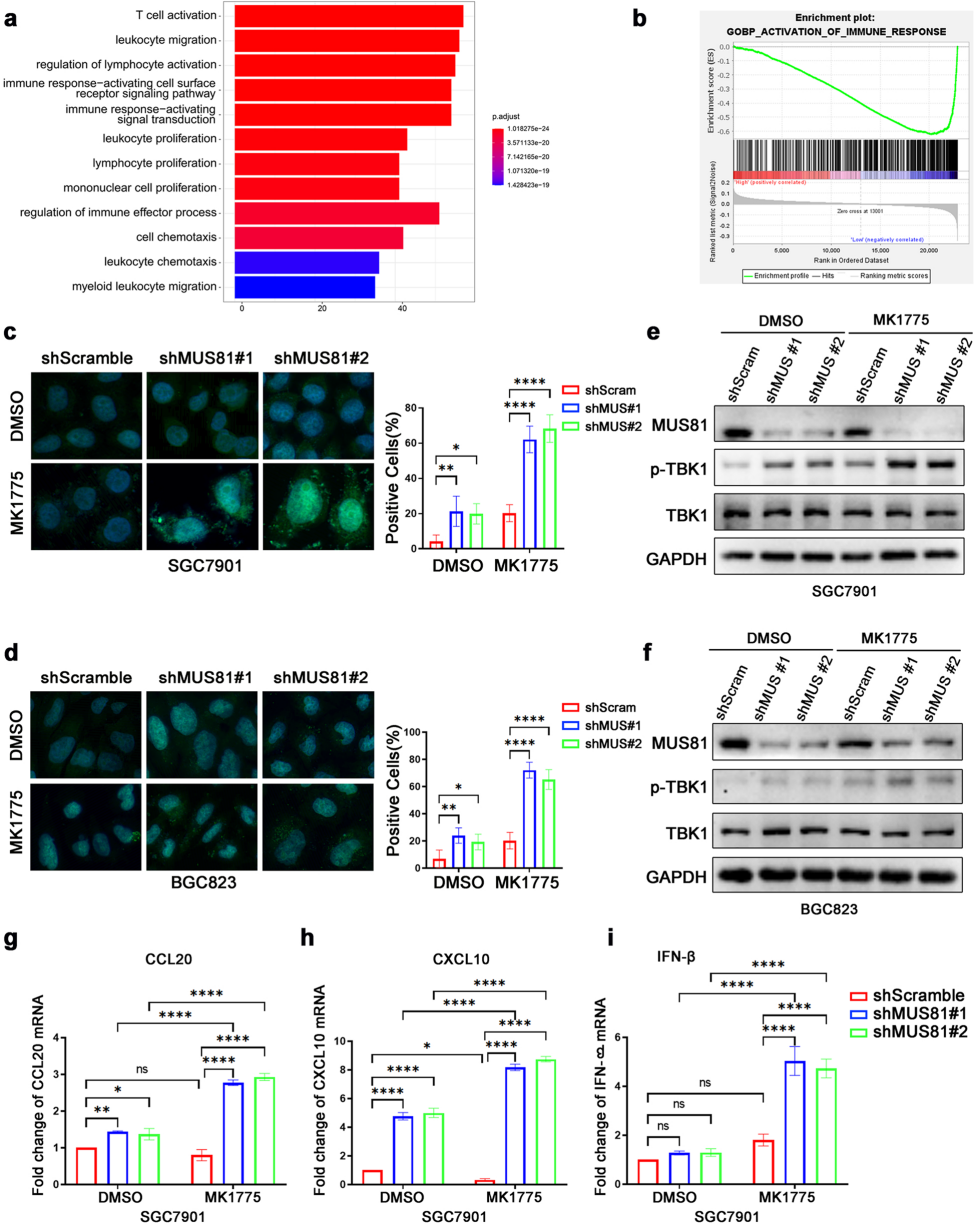
MUS81 inhibition amplifies cytosolic DNA accumulation and promotes innate immune activation in gastric cancer cells. **a** Gene Ontology analysis of MUS81-related differentially expressed genes (DEGs) in the GSE62254 data set. **b** Gene Set Enrichment Analysis of MUS81-related DEGs during immune response. *P* = 0.002, FDR q-value = 0.048. **c** and **d** Representative images of PicoGreen staining in shScramble or MUS81 knockdown (shMUS81#1 and shMUS81#2) SGC7901 and BGC823 cells treated with dimethyl sulfoxide (DMSO) or MK1775 (0.50 μM for SGC7901 and 0.25 μM for BGC823). Data are reported as mean ± SD for five independent experiments. **e** and **f** Western blot of phosphorylated TBK1 (p-TBK1) and total TBK1 treated with MK1775 (0.50 μM) in SGC7901 and MK1775 (0.25 μM) in BGC823 cells for 24 h. **g**–**i** qPCR of proinflammatory cytokines CCL20 and CXCL10 and IFN-β in SGC7901 cells treated with MK1775 (0.50 μM) for 12 h. The data are presented as mean ± SD (n = 3). * *P* < 0.05; ** *P* < 0.01; *** *P* < 0.001; **** *P* < 0.0001; *ns.*, not significant

Next, we analyzed the potential function of MUS81-related DEGs to evaluate the further therapeutic value of targeting MUS81. The GO enrichment analysis showed that MUS81 might play an essential role in the immune response (Fig. 4a), and Gene Set Enrichment Analysis showed that MUS81 was negatively correlated with activation of the immune response in gastric cancer (*P* = 0.002, FDR q-value = 0.048) (Fig. 4b). Previous studies have reported that several drugs targeting the DDR network increased the accumulation of cytosolic dsDNA, which could be recognized by the STING pathway, thus inducing the phosphorylation of TBK1, leading to the secretion of proinflammatory cytokines and activation of the innate immune response. Therefore, we investigated whether MUS81 targeting could amplify the cGAS/STING signaling activation induced by WEE1 inhibition. As expected, we observed an elevated cytosolic dsDNA accumulation in MUS81 knockdown gastric cancer cells (Fig. 4c and d). We also found that MK1775 triggered the phosphorylation of TBK1 efficiently in MUS81 knockdown gastric cancer cells (Fig. 4e and f). In addition, MK1775 treatment significantly increased the expression of proinflammatory cytokines such as CCL20, CXCL10, and IFN-β in MUS81 knockdown cells, but increased expression modestly in parental gastric cancer cells (Fig. 4g–i). These data demonstrated that targeting MUS81 triggered the activation of cGAS/STING signaling induced by MK1775 treatment in gastric cancer cells.

### WEE1 inhibitor MK1775 promotes the CD8^+^ T cell activation induced by MUS81 knockdown

**Fig. 5:**
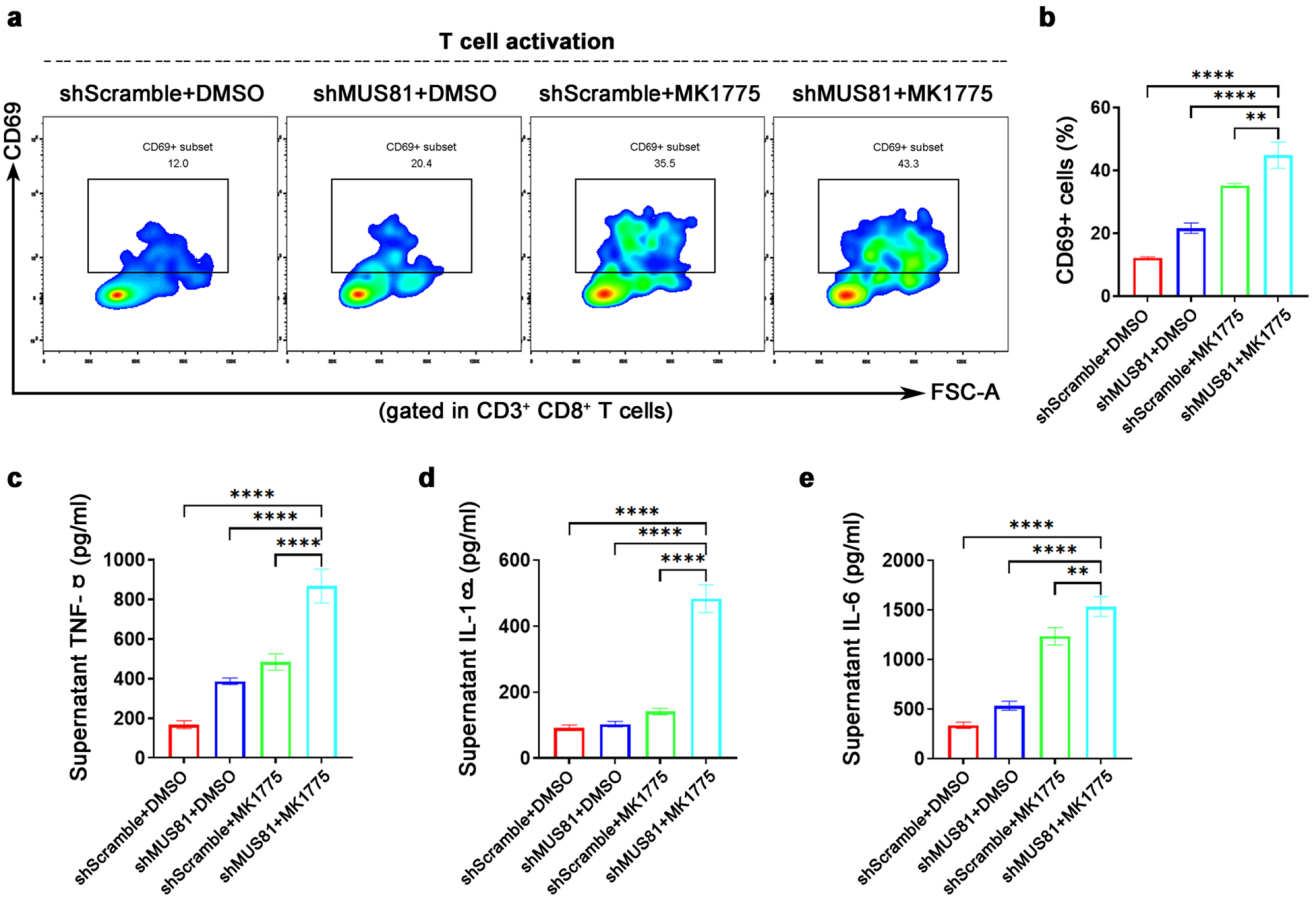
WEE1 inhibition enhances the CD8^+^ T cell activation induced by MUS81 knockdown. **a** Representative images of CD8^+^ T cells activation after co-cultures. CD69^+^ cells were gated in CD3^+^/CD8^+^ T cells. MFC shScramble or shMUS81 cells were treated with dimethyl sulfoxide (DMSO) or MK1775 (1.0 μM). **b** Statistics analysis of the proportion of CD69^+^ cells to CD3^+^/CD8^+^ T cells in the indicated four groups. The data are presented as mean ± SD (n = 3). **c**–**e** ELISA analysis of TNF-α (left), IL-1β (middle), and IL-6 (right) in the supernatants of the co-cultures. The data are presented as mean ± SD (n = 3). * *P* < 0.05; ** *P* < 0.01; *** *P* < 0.001; **** *P* < 0.0001; *ns.*, not significant

We conducted a co-culture experiment *in vitro* to determine the activation state of T cells to explore the effect of MUS81 knockdown and MK1775 on innate immunity. First, we extracted and induced mouse bone marrow DCs. MK1775 (1.0 μM) was used to treat mouse gastric cancer MFC cells for 48 h, and then, MFC cells were added to DCs at a ratio of 1:10. After 24 h of co-cultivation, DCs were harvested and co-cultured with mouse spleen lymphocytes at a percentage of 1:10. Finally, after 72 h of co-cultivation, cells were harvested and labeled with CD3, CD8, and CD69, and the proportion of CD69^+^ T cells was analyzed by flow cytometry analysis. As shown in Fig. 5a and b, the percentage of CD69^+^ T cells in the shMUS81 group was significantly elevated after MK1775 treatment, indicating that MK1775 can enhance shMUS81-induced CD8^+^ T cell activation. In addition, we performed ELISA on the supernatant after co-cultivation. The supernatant of the combination treatment group contained significantly higher levels of TNF-α (Fig. 5c), IL-1β (Fig. 5d), and IL-6 (Fig. 5e) than those of the other three groups.

### WEE1 inhibitor MK1775 enhances the anticancer effect of ICB therapy in MUS81 deficient gastric cancer

**Fig. 6:**
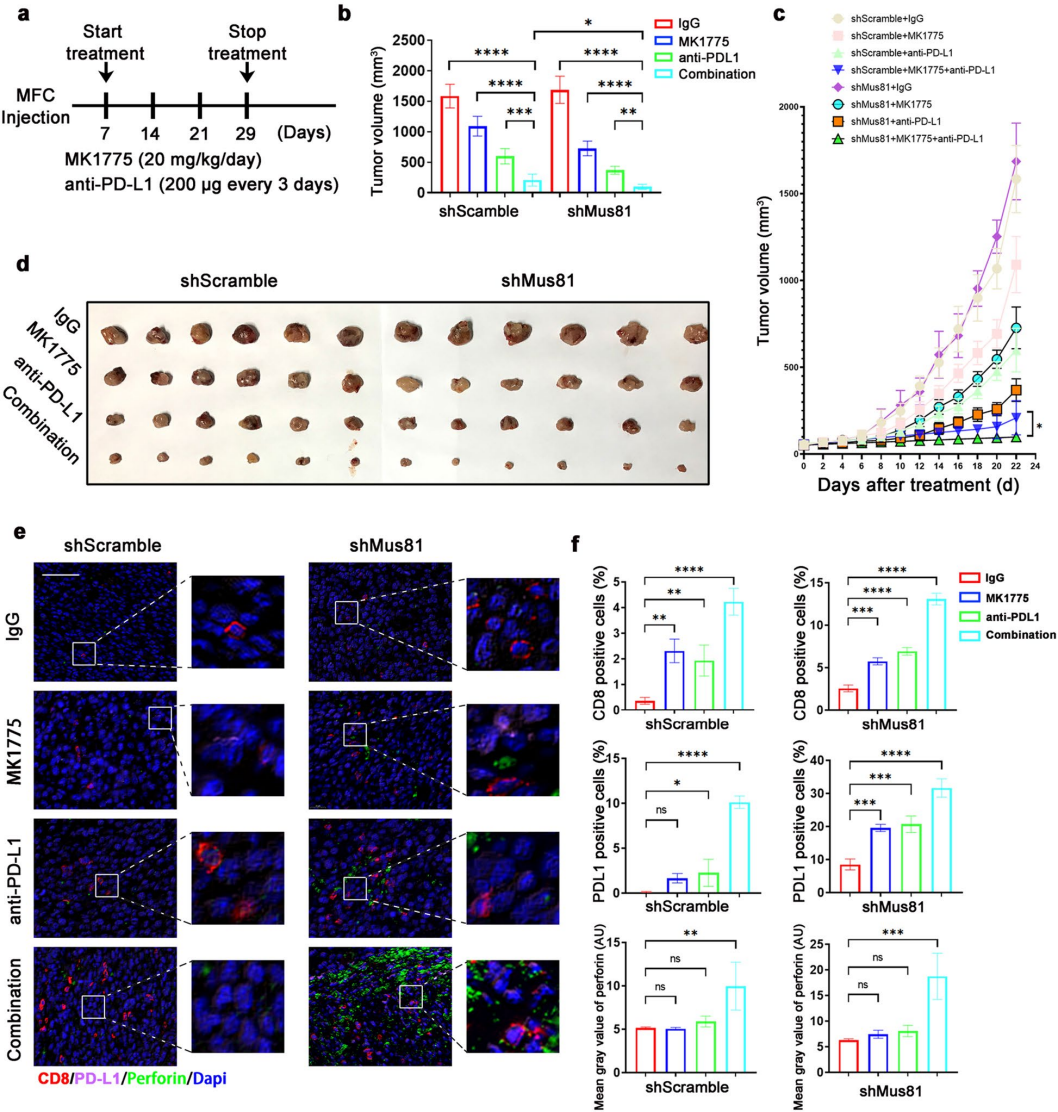
WEE1 inhibitor MK1775 enhances the anticancer effect of immune checkpoint blockade (ICB) therapy in MUS81 deficient gastric cancer cells. **a** Schematic of isotype control IgG, MK1775, anti–PD-L1 antibody and combination treatment. Treatments were started on day 7 after inoculation and stopped on day 29. **b** Tumor volume of the indicated groups on day 29. Data of tumor volume are represented as mean ± SD (n = 6 for each group). **c** Tumor growth curve of indicated groups. Data of tumor volume are represented as mean ± SD in indicated groups (n = 6 for each group). **d** Images of tumor of MFC gastric cancer mice model. **e** multiplexed immunofluorescence (mIF) staining of CD8 (red), perforin (green), and PD-L1 (pink) in parental and MUS81 knockdown MFC tumors. Scale bar: 50 μm. **f** Quantitative analysis of CD8 and PD-L1 positive cells and the fluorescence intensity of perforin. Top, proportion of CD8 positive cells in MFC shScramble (left) or shMUS81 (right) tumors. Middle, proportion of PD-L1 positive cells in MFC shScramble (left) or shMUS81 (right) tumors; Bottom, the fluorescence intensity of perforin in MFC shScramble (left) or shMUS81 (right) tumors. The data are presented as mean ± SD (n = 3). * *P* < 0.05; ** *P* < 0.01; *** *P* < 0.001; **** *P* < 0.0001; *ns.*, not significant

**Fig. 7:**
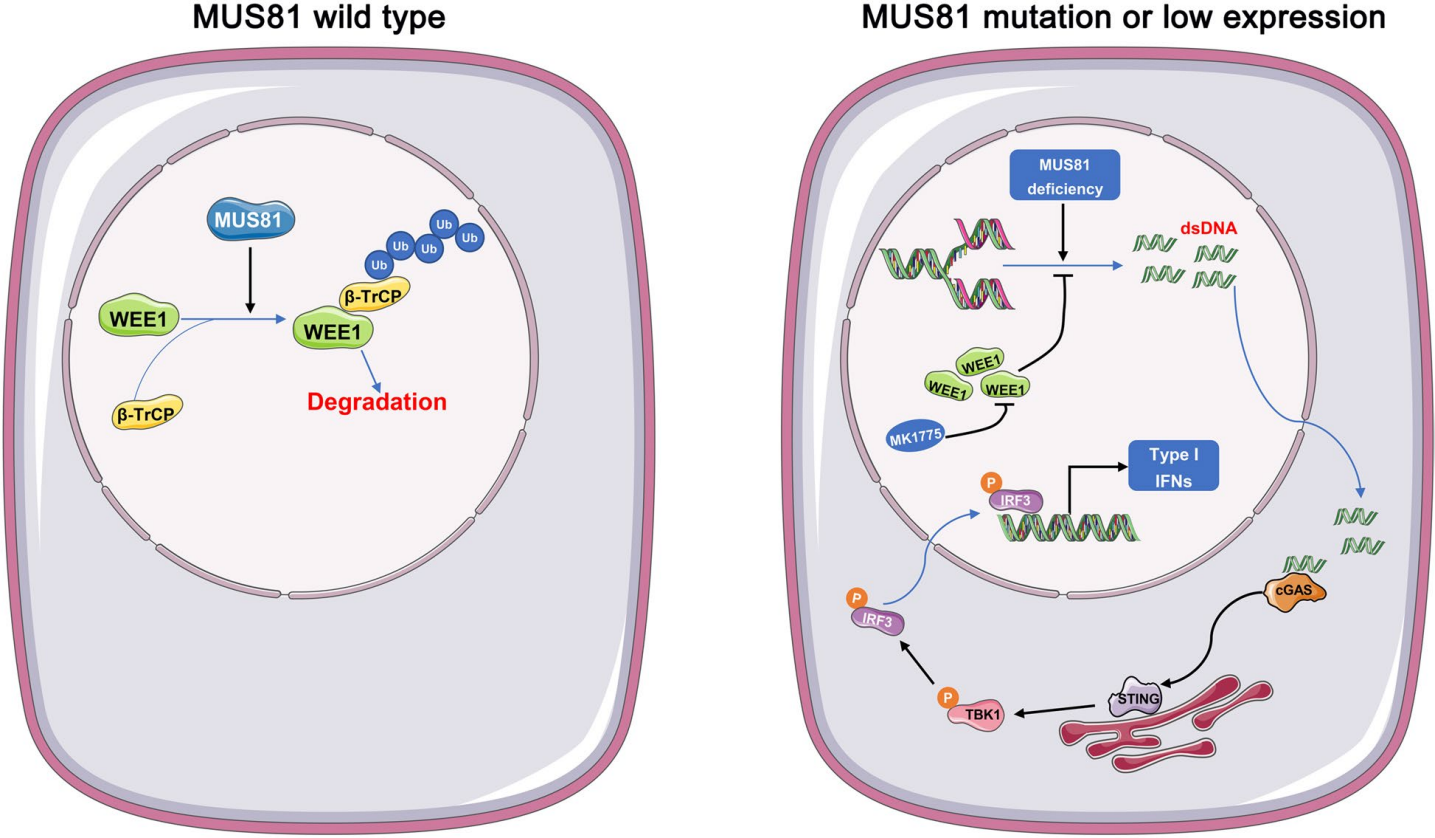
Schematic of MUS81 regulating WEE1 kinase and the anticancer effect of WEE1 inhibitor and immune checkpoint blockade (ICB) combination therapy in gastric cancer

Furthermore, using the MFC gastric cancer syngeneic mouse model we evaluated whether targeting MUS81 promoted the anticancer effect of MK1775 combined with ICB treatment in gastric cancer. Seven days after cancer cell injection, mice were treated with MK1775 daily and with PD-L1 antibody every 3 d (Fig. 6a) for a total of 22 d. Although combination therapy presented a good anticancer effect in the parental group, MUS81 knockdown not only sensitized the anticancer effect of both monotherapies but also vastly amplified the therapeutic effect of the combination therapy (Fig. 6b–d). Furthermore, multi-immunofluorescence staining (Fig. 6e) analysis indicated that compared with monotherapy or parental groups, the combination therapy remarkably increased CD8^+^ T cell infiltration and perforin in MUS81 knockdown cancer cells (Fig. 6f). In addition, an increased proportion of PD-L1 positive cells was also observed in the combination therapy group (Fig. 6f). In summary, we provided reliable data to demonstrate that MUS81 targeting amplified the activation of innate immune response and promoted the anticancer effect of WEE1 inhibitor and ICB combination therapy in gastric cancer (Fig. 7).

## Discussion

Although immune checkpoint inhibitors such as PD-1 and PD-L1 inhibitors trigger anticancer immune responses, which have been proven useful in the clinical treatment of a series of cancer types [28–30], the application of these reagents is severely restricted in gastric cancer [31, 32]. Previous studies linked DNA damage with innate immunity, which could enhance the therapeutic effect of ICB therapy; therefore, elucidating the interplay between the DDR network and innate immunity can provide a novel strategy for cancer treatment [33, 34]. Our study found that high MUS81 expression indicates poor prognosis in gastric cancer and that targeting MUS81 elevates the expression of WEE1 owing to the disruption of β-TRCP-induced ubiquitination. Furthermore, MUS81 inhibition increases replication stress and impairs replication stress-associated DNA repair, thereby accumulating cytosolic DNA in gastric cancer cells and activating cGAS/STING signaling. Although the WEE1 inhibitor MK1775 partly enhanced the anticancer effect of PD-L1 inhibitors by activating the cGAS/STING pathway, MUS81 targeting further amplified the anticancer effect of this combination strategy because of the elevated cytosolic dsDNA in gastric cancer cells.

MUS81 is an essential structure-specific endonuclease, which has an evolutionarily conserved role from yeast to human beings and has a significant effect on DDR, DNA replication stress, and genomic integrity maintenance [21, 35]. Previous studies have presented different phenotypes of carcinogenesis with MUS81 knockout and have reported a similarity in the expression level of MUS81 in different malignant diseases, which provides a basis for the hypothesis that the role and therapeutic potential of MUS81 are intertwined [36, 37]. In this study, we observed that MUS81 knockdown led to the accumulation of cytosolic DNA and STING pathway activation in gastric cancer, which was contrary to the previously reported phenotype in prostate cancer [38]. This finding supports our hypothesis. MUS81 might act as a tumor suppressor in cancers with low expression levels and as an oncogene-like molecule in those tumors with high MUS81 expression levels [38]. Previous studies have provided preliminary evidence that targeting MUS81 could sensitize several chemotherapeutic drugs in cancer cells [39, 40]; in addition, our previous study reported that MUS81 could serve as a therapeutic target for the BRD4 inhibitor AZD5153 via regulation of Sirt5 [26], although its therapeutic potential needs to be further discussed in gastric cancer. Our study showed that high expression of MUS81 was linked with poor prognosis, and MUS81 expression was negatively correlated with the expression of WEE1 kinase, a key player in cell cycle regulation [41]. In line with the bioinformatics analysis, MUS81 knockdown elevated the expression of WEE1. We observed a decrease in WEE1 levels in cells overexpressing the wild-type MUS81 plasmid but not in cells with overexpression of the enzymatic activity mutant plasmid, indicating that MUS81 regulated the expression of WEE1 in an enzymatically dependent manner. Notably, we further identified a novel role of MUS81 in regulating the ubiquitination of WEE1. Although β-TRCP has been recognized as an E-3 ligase that can bind with WEE1 to promote its ubiquitination and regulate the abundance of DNA damage response-related proteins at DNA damage sites [42], it remains unclear how β-TRCP-regulated ubiquitination is processed. Our study demonstrated that MUS81 regulated the function of β-TRCP and altered its targeting capacity, thus decreasing the binding of the β-TRCP-WEE1 complex via MUS81 enzymatic activity. The mechanism underlying this phenotype is MUS81 loss, which causes conformational changes in the β-TRCP complex, which in turn may impair the capacity of β-TRCP to bind with WEE1 and promote its deubiquitination.

Abnormalities in DNA damage repair commonly occur in malignant diseases, and the balance of DDR signaling sometimes goes for a toss [43]. MUS81 inhibition could elevate the expression of WEE1 kinase, indicating that WEE1 plays a vital role in DDR and genomic stability maintenance in MUS81-deficient cells. Moreover, this may provide us a rational explanation as to why MUS81 inhibition alone had a limited effect on the proliferation of gastric cancer cells. The DDR network is complicated and the activation of other DNA repair pathways might compensate and overcome the endogenous DNA damage in MUS81-deficient cells; thus, MUS81 deficiency only impaired cell proliferation in cells with a specific gene mutation, such as the BRCA2 mutation [44], and these mutations are rare in gastric cancer. In the current study, we demonstrated that MK1775 increased the accumulation of cytosolic dsDNA and activation of TBK1 phosphorylation in MUS81-deficient gastric cancer cells, indicating that MK1775 could activate the innate immune response via the cGAS/STING pathway. STING activation promotes the transcription and expression of type I interferons, mainly IFN-α and IFN-β. Therefore, we investigated type I interferon target genes such as *CCL5, CXCL10*, and *CCL20* and other classical pro-inflammatory cytokines such as TNF-α, IL-1β, and IL-6. Our results showed that only *CXCL10, CCL20*, and *IFN-β* exhibited significant changes, although limited changes were observed for the rest. In addition, interfamilial cell co-culture showed that MK1775 exposure in MUS81 knockdown gastric cancer cells increased the early activation of CD8^+^ T cells. This finding also provides a mechanistic basis for targeting MUS81 to enhance the therapeutic response of the WEE1 inhibitor and ICB combination therapy in gastric cancer. Cytotoxic T cell activation and infiltration is a crucial process during ICB anticancer immune response, and STING pathway activation induced by endogenous and exogenous stimulation could increase the number of tumor-infiltrating lymphocytes and promote the survival and infiltration of memory T cells in the tumor microenvironment [44, 45]. We found that MK1775 and ICB combination treatment significantly elevated CD8^+^ T cell infiltration and perforin expression in the MUS81 deficient gastric cancer immune proficient mouse model compared to other groups, indicating increased cytotoxic T cells in the tumor microenvironment. Notably, elevated PD-L1 positive cells were observed in the combination treatment groups or monotherapy groups, and this finding was similar to the results of a previous study [46]. In addition, cytokines such as CCL5 and CXCL10 may promote cancer cell proliferation by recruiting myeloid cells, and this is essential to activate the immune checkpoint PD-1/PD-L1 axis, which establishes an immunosuppressive tumor microenvironment in multiple cancer types [47–50]. A significant increase in the number of PD-L1 positive cells indicates that combination treatment may recruit myeloid cells into tumor sites to counterbalance the therapeutic efficacy, which provides the possibility to strengthen the anticancer effect of combining MK1775 and anti-PD-L1 by targeting myeloid cells in the microenvironment of MUS81 deficient gastric cancer.

Based on previous studies, ICB monotherapy seems to provide limited benefits for patients with advanced gastric cancer. Targeting the DNA damage response network also presented low therapeutic efficacy and intolerable toxic effects regardless of monotherapy or combination therapy [51, 52]. The emerging concept that DDR reagents or radiotherapy can activate cancer cell innate immunity to enhance the therapeutic effect of ICB provides a novel strategy for cancer treatment [53]. Our study presents a new approach of targeting crux DDR protein loss-induced molecular vulnerabilities conferred by alterations in the DDR network. Our study showed that the anticancer effect of WEE1 inhibitor and combination therapy with PD-L1 antibodies are both significantly enhanced in MUS81 deficient cells, which suggests that inhibition of WEE1, or a broad inhibition of the DDR network, may require synergistic association with defects in DDR pathways to achieve optimal immune-modulating effects.

## Conclusions

Our study reports that MUS81 targeting could enhance the immune-modulating effect of WEE1 inhibitors, and this might promote new strategies and overcome obstacles during clinical treatment of patients with advanced gastric cancer.

## Supplementary Information

**Additional file 1: Table S1**

**Additional file 2: Table S2**

**Additional file 3: Table S3**

## Data Availability

The analyzed datasets in the study are available from public datasets or the corresponding authors on reasonable request.

## Abbreviations

cGAS: Cyclic GMP-AMP synthase
DCs: Dendritic cells
DDR: DNA damage and repair
DEGs: Differentially expressed genes
ICB: Immune checkpoint blockade
MUS81: Methyl methanesulfonate and ultraviolet-sensitive gene 81
EMT: Epithelial-mesenchymal transition
qRT-PCR: Quantitative reverse transcription polymerase chain reaction
IHC: Immunohistochemistry
GEO: The Gene Expression Omnibus database
OS: Overall survival
GO: Gene Ontology
